# Urban Green Spaces, Greenness Exposure and Species Richness in Residential Environments and Relations with Physical Activity and BMI in Portuguese Adolescents

**DOI:** 10.3390/ijerph18126588

**Published:** 2021-06-18

**Authors:** Juliana Melo, Ana Isabel Ribeiro, Susana Aznar, Andreia Pizarro, Maria Paula Santos

**Affiliations:** 1 Research Center in Physical Activity, Health and Leisure (CIAFEL), Faculty of Sports, University of Porto (FADEUP), Rua Dr. Placido Costa, 91, 4200-450 Porto, Portugal; ana.isabel.ribeiro@ispup.up.pt (A.P.); msantos@fade.up.pt (M.P.S.); 2 EPIUnit, Instituto de Saúde Pública da Universidade do Porto, Rua das Taipas 135, 4050-600 Porto, Portugal; gabrtl@fade.up.pt; 3 Laboratory for Integrative and Translational Research in Population Health (ITR), Rua das Taipas 135, 4050-600 Porto, Portugal; 4 PAFS Research Group, Faculty of Sports Sciences, University of Castilla-La Mancha (UCLM), Avda. Carlos III s/n, 45071 Toledo, Spain; susana.aznar@uclm.es; 5 Biomedical Research Networking Center on Frailty and Healthy Aging (CIBERFES), 28029 Madrid, Spain

**Keywords:** overweight, pediatric obesity, physical activity, built environment, public green space, biodiversity

## Abstract

Environmental factors play an important role in obesity-related behaviors. Evidence indicates significant associations between weight and urban green spaces in adults, but it is not clear whether this relationship applies to adolescents. Therefore, our aim was to determine the associations between urban green spaces, greenness exposure and species richness in residential environments with physical activity and body mass index. Sixty-two adolescents between 12 and 18 years of age answered a self-administered questionnaire, providing information on height, weight, age, sex and home address. Data on socioeconomic deprivation were obtained from the European Index of Deprivation for Small Portuguese Areas. Physical activity levels were assessed using accelerometers. Urban green space counts and the normalized difference vegetation index values were measured using buffers along the roads with distances of 300, 500, 1000 and 1500 m from each participant’s residence. To quantify the species richness, the species richness index was used. Linear regression models were fitted to analyze whether urban green spaces, exposure to green spaces and species richness counts for each distance were associated with physical activity and self-reported body mass index. We did not find significant associations between the independent variables and the probability of overweight or obesity. The relationship between environmental variables, adolescents’ physical activity and body weight seems to be complex and further studies may contribute to better understanding of the topic.

## 1. Introduction

In recent decades, obesity and overweight have shown rapid increases and high prevalence in children and adolescents. This scenario has amplified the concern for the health of young people worldwide and has motivated researches to clarify causal factors of obesity for these age groups [1].

A high body mass index (BMI) is strongly associated with an increased risk of developing chronic diseases, such as cardiovascular diseases [2], diabetes mellitus [3], cancer [4] and some musculoskeletal disorders [5]. In addition to a greater risk of developing chronic non-communicable diseases, overweight or obesity during childhood tends to persist into adulthood [6,7].

Several studies have shown that children and adolescents who engage in more physical activity (PA) have a lower BMI than those who are less active [8,9]. In this context, physical inactivity and sedentary behaviors are increasingly being identified as determining factors for pediatric overweight or obesity [10,11].

In this context, socioecological models suggest that individual, interpersonal, environmental and political factors influence health and PA behaviors [12]. The main theory of socioecological models is that influences at various levels can interact and exert synergistic effects on behaviors, and in this sense interventions at various levels may be more effective for changing behaviors [13]. Environmental characteristics, such as access to recreation facilities, aesthetics and infrastructure for pedestrians, seem to consistently predict general and recreational PA, while neighborhood mobility and street connectivity seem to be consistently related to PA for transportation [14].

Recent studies have demonstrated that certain characteristics of the built environment, such as street connectivity and transport systems, are capable of promoting PA; therefore, environmental interventions seem to be a sustainable strategy to encourage young populations to engage in or increase PA [15]. Moreover, the influence of urban environment seems to be a good strategy when designing future community-based interventions to promote more active lifestyles among adolescents, particularly when adolescents’ self-efficacy and moderate-to-vigorous physical activity (MVPA) are low [16].

As the existing studies did not simultaneously consider the impacts of all the characteristics of the built environment, it is still difficult to indicate the characteristics that most affect the levels of PA of adolescents [17]. Regarding the perceived barriers, it is known that a lack of motivation [18], lack of time [19], lack of knowledge of the existing PA facilities or program, lack of transportation, concerns with neighborhood security and lack of access to recreational facilities are often reported [20]. Regarding studies that have used measures of the built environment, most of them have reported inconsistent associations with street connectivity and good walkability with PA but consistent relationships between the availability of recreational facilities and mixed land use [21].

Among recreational facilities, urban green spaces (UGS) in particular have been attracting attention [22]. This type of space is an easily modifiable element for the promotion of PA and the reduction of sedentary behaviors. 

The relationship between PA and BMI is well documented and the presence of UGS in the residential environment has been increasingly described as one of the determinants highly associated with overweight and obesity [8,9].

The negative relationship between the number of UGS in the residential area BMI has been consistently reported among child populations [23,24]; however, few studies have investigated this connection among adolescents and there is insufficient evidence to make consistent associations [25]. 

Although many studies have demonstrated positive impacts of UGS on human health and well-being [26,27], it is not clear whether solutions based on an increase in urban nature can really contribute positively to increase PA levels and consequently reduce in overweight and obesity levels [28,29].

Given this scenario, green space measures, such as exposure to green spaces and biodiversity, can be assessed to help understand and comprehensively clarify the possible relationships between UGS and health [30].

We aimed to explore whether the number of UGS, the level of green exposure and the amount of species richness in the residential environment were associated with PA and BMI in Portuguese adolescents, and whether these relationships change with adjustment to the socioeconomic deprivation index.

## 2. Materials and Methods 

### 2.1. Study Area

Oporto is the second city and the fourth most populous municipality in Portugal. The municipality, with an area of 41.42 km^2^ [31], has a population of around 237,000 inhabitants within its administrative limits, being subdivided into seven parishes [32]. 

### 2.2. Study Design

Data were collected in 4 of the 7 public high schools in Oporto city. The schools were selected for convenience, according to the interest of teachers and school managers in participating in the study. Participation was requested from all adolescents enrolled in physical education classes and whose teachers agreed to participate in the research.

### 2.3. Sample

The calculations for determining the sample size with a confidence level of 95% and a margin of error of 5% resulted in a total of 382 subjects [33].

We obtained a total of 311 participants residing in the metropolitan area of Porto; however, since data referring to the species richness index were only available for the city of Porto, 249 subjects were excluded because they lived outside the limits of this municipality. The final sample size comprised 62 adolescents (32 girls, 30 boys) between 12 and 18 years of age. Prior to data collection, both participants and their parents received a complete explanation of the purpose of the investigation and signed a written consent form to participate in the study. 

Ethical approval for this study was obtained from the Ethic Committee of the Faculty of Sports of the University of Porto (CEFADE 22.2013). Consent was also collected from the Education Authority and the School Board.

### 2.4. Individual Anthropometric and Sociodemographic Data

Information on height, weight, age, gender and residential address were obtained through a self-administered questionnaire. 

The measure commonly used to define the weight status is the BMI and associations between BMI and health outcomes within and between populations are often used to help determine possible causes of illness [34]. 

The BMI values were calculated as weight in kilograms divided by height in meters squared (kg/m^2^), while the BMI z-scores were categorized into normal weight (1 standard deviation (SD)), overweight (>+1SD) and obesity (>+2SD) [34]. For analysis purposes, the overweight and obesity categories were grouped into just one category.

### 2.5. Socioeconomic Deprivation

Data regarding the socioeconomic deprivation of the sample subjects were obtained from the European Index of Deprivation for Small Portuguese Areas (EDI-PT) [35].

The EDI-PT is a sensitive measure to capture health inequalities, as it is associated with the development and health of the population [35]. The present variable was categorized into terciles (1 for least private and 3 for more private), which were then classified as “low”, “medium” and “high” deprivation, respectively. This strategy aimed to maximize the heterogeneity of results [36].

### 2.6. Physical Activity

Physical activity (PA) levels were assessed using the Actigraph model GT3X+ accelerometers (Actigraph, Pensacola, FL, USA). Participants used an accelerometer attached to an elastic belt positioned on the right side of the pelvic girdle throughout waking hours for seven consecutive days, except during bathing and water activities.

The epoch was set to 30 s, and for data analysis purposes only participants with a minimum of 600 min of daily use for 4 weekdays and 1 weekend day (or with 3000 min of use over 4 days) were included [37].

The cut-off points proposed by Evenson [38] were used to categorize moderate-to-vigorous PA (MVPA). 

### 2.7. Assessment of the Geographical Accessibility of Urban Green Spaces 

A total of 226 public green spaces were considered, with no restrictions on size, location or characteristics, covering the universe of public green spaces available in the study area that could be freely (free of charge) used by the population. Polygons of green spaces and entrance places were obtained from digital city maps, as described in previous studies [39].

Using the location of each child’s residence as a starting point, the following measures based on a network of geographic accessibility to green spaces were calculated: number of available green spaces within 300, 500, 1000 and 1500 m from the residence and school (score). When the green spaces were delimited (that is, with fences), we used the distance to the entrance; otherwise, the distance to the limit was used.

For the calculations, we used ArcGIS software version 10.5 and the Network Analyst extension, using an updated street network dataset provided by the Environmental Systems Research Institute.

### 2.8. Normalized Difference Vegetation Index

The normalized difference vegetation index (NDVI) was used to analyze the greenness exposure [40]. To capture the surrounding greenery, we calculated the average NDVI using buffers along the road with distances at 300, 500, 1000 and 1500 m from each participant’s residence. The NDVI is calculated based on the ground surface reflectance of the visible red (VISR) and near-infrared (NIR) wavelengths using the formula presented in Equation (1):

NDVI = (NIR − VISR)/(NIR + VIRSR)(1)

The underlying principle employed in the NDVI calculation is that chlorophyll in healthy vegetation absorbs radiation in the VIRS region (630–690 nm) of the electromagnetic spectrum and reflects radiation in the NIR region (760–900 nm). It is a unitless index ranging from −1 to 1, with higher values corresponding to a higher density of healthy vegetation. For this study, images with 5% or less of Landsat 8 cloud cover (spatial resolution: 30 m) were used during the spring–summer (vegetation peak) period of 2016–2017 [41]. 

ArcMap 10.5 was used to process satellite images and QGIS 3.8.2 was used to extract the average NDVI for each participant.

### 2.9. Species Richness Assessment

In order to quantify the species richness, we used the species richness index (SRI). This index was obtained from a species richness report previously compiled by Porto City Council and the Research Center for Biodiversity and Genetic Resources (CIBIO) [42,43]. The number, type and area of habitats in Porto were retrieved, followed by an estimation of the number of species living in those habitats. Included species consisted of four vertebrate groups (amphibians, birds, reptiles and small mammals), totaling 89 different species. 

To express the concentrations of species in the habitats that surrounded the residences, we summed the numbers of different species present in the habitats located within different network-based buffers (300, 500, 1000 and 1500 m) multiplied by the total area of each habitat, then subsequently we divided the resulting values by the buffer area in square meters. 

Such an approach has been used in previous studies, such as the EXALAR XXI project (POCI-01-0145-FEDER-030193; https://www.exalar21.com/home|accessed on 13 November 2019) [44,45]. 

### 2.10. Statistical Analysis

Adjusted linear regression models were used to analyze whether UGS counts, exposure to green spaces (NDVI) and SRI for road distances of 300, 500, 1000 and 1500 m were associated with PA and BMI. The models were applied to the entire sample and by gender. The EDI-PT of the residential neighborhood was included in the model as an adjustment variable.

The likelihood ratio tests were performed to compare the power of a predictive model. Collinearity diagnostics were run on the variables included in each model. Odds ratios were calculated for 95% confidence intervals.

The intraclass correlation coefficient (ICC) was also calculated to provide a better understanding of the proportion of variance due to group differences [46].

All statistical analyses were performed using Statistical Package for the Social Sciences (SPSS) version 25 (IBM Corp, Armonk, NY, USA).

## 3. Results

The sample characteristics are presented in Table 1. About 51% of the students were girls. The sample deprivation index values were predominantly (71.1%) medium and high. The BMI average for girls was 21.7 ± 3.6 and for boys was 22.2 ± 2.9 kg/m^2^. About 83% of the adolescents did not achieve daily PA recommendations of 60 min of MVPA, with the average MVPA being about 41 min.

Descriptive statistics for each variable by gender and by BMI categories are shown in Table 2. About 23% (*n* = 14) of the sample was overweight or obese. The average BMI for the normal weight group was approximately 20 kg/m^2^ for both sexes. 

In relation to the UGS average at 300 m, the residential environment for boys with normal weight contained fewer green spaces when compared with the environment for boys with overweight / obesity (*p* = 0.04).

In this study, the ICC value (ICC = 0.08) suggested that clustering between neighborhoods and schools was non-existent; therefore, a multilevel analysis was not used [46].

The crude regression models performed individually with each predictor (Tables S1–S3, S6–S8) did not show significant results. Models including all predictors simultaneously did not show significant results (see Table 3). In addition, we tested the model stratified by gender (see Tables S4, S5, S9 and S10) and found that the socioeconomic deprivation index was inversely correlated to BMI in boys (r = −0.4), but the models result did not show statistical significance.

Since socioeconomic deprivation seems to play an important role in the relationship with overweight and obesity, we developed a model with adjustment for the socioeconomic deprivation index (see Table 4); however, the results were the same, showing no significant statistics.

Likelihood ratio tests showed that the addition of variables to each subsequent model did not significantly improve the model fit. Collinearity diagnostics did not reveal major concerns, with no variance inflation factor scores above three.

## 4. Discussion

An increasing number of studies have sought to assess the relationship between urban nature and human health, using many different research approaches and obtaining heterogeneous results.

In our study, the socioeconomic deprivation index was inversely related to BMI in boys and no significant results were found for the other variables. Potential confounding variables were included but the size of the effect did not change. Regardless, our findings raise some interesting points for discussion.

There is a general consensus that green spaces close to residential areas support human health and that their distance to a subject’s home is important [47].

The European Environment Agency (EEA) [48] recommends a walk of up to 15 min to the nearest UGS. Nielsen and Hansen [49] claim that a distance of 300 to 400 m is seen as a limit value, after which the frequency of use begins to decrease. However, Kaczynski, Besenyi [50] demonstrated that the existing recommendations do not appear to be relevant for adolescent populations. Ding and Gebel [51] corroborated this hypothesis when reporting that the proximity of the UGS was not associated with PA in adolescents; in addition, they concluded that the use of objective measures increased the consistency of the results.

In this context, McCrorie et al. [52] stated that part of the recent growth in the PA environmental determinants can be attributed to the advancement of technology. The use of objective approaches enables accurate measurements, allowing a more detailed representation of the environment. 

In particular, the objective measures of PA, accessibility to the UGS and exposure to green spaces seem to be very relevant with regard to the identification of environmental determinants about the use of UGS and the relationship with the health of populations [52,53,54].

Except for the weight and height obtained by self-report, in our study we mainly used information from objective measures, with the latter method seeming to be more advantageous compared to the subjective measures; thus, we consider that our results are likely to be quite reliable, although we emphasize that the environmental determinants related to UGS in favor of health seem complex and interrelated [55].

In addition, despite the unanimity of the benefits of UGS for health populations, certain environmental determinants of the use and provision of UGS may not be universal, since each city has a specific structure and a peculiar group of inhabitants [56]. 

Although UGS have generally been treated as a type of homogeneous environment, some studies have separated the spaces into relatively broad typologies, suggesting that some types affect health to varying degrees; that is, not all UGS are equal in their health benefits [57]. 

The composition and configuration of urban green spaces are usually measured through metrics of the landscape [11]. Among the dozens of existing methods, the NDVI has been gaining prominence in studies that analyze green spaces and their relationship with health [58].

Although some studies have postulated that more green closer to home may be important for health [59], in our study we did not find any significant associations between NDVI, PA and BMI in adolescents.

Recently Ekkel and de Vries [60] conducted a review study on green spaces and health, showing that associations vary within and between studies, mainly due to the different buffer sizes, a factor that probably ends up leading to an inconsistent direction of the effects.

Eventually, if similar approaches are replicated elsewhere, they may be able to provide more information about the mechanisms by which exposure to green spaces may or may not influence PA and BMI in juvenile populations.

In this context, the relationship between UGS and health is becoming prominent in the urban planning agenda and in development policies, which already refer to the positive effects of the presence, use and design of UGS [29,56].

Despite the UGS being integrated into policies and urban planning development, the role of these spaces in supporting biodiversity and the links between biodiversity and human health have received insufficient attention, with the few existing studies on these subjects presenting inconclusive results [61].

This study was one of the first to explore the influence of species richness on adolescents’ PA and BMI, for which we found no significant results.

Interestingly, the results from the study by Schwartz et al. [62] highlighted that the loss of urban biodiversity can result in compromised ecosystem functions, which in turn can negatively influence human health.

In contrast, the study by Leemans and De Groot [63] concluded that a decline in ecosystems was accompanied by steady gains in human well-being at the global level. Using this logic, Hough [64] explained that this discovery seems intuitive, since economic or industrial development tends to threaten species and at the same time increases human life expectancy.

However, Mills et al. [65] found that industrialized urban habitats are poor in biodiversity, which ends up discouraging contact with beneficial environmental microbiotics for the human body. When associated with inadequate diets, excessive intake of antibiotics, physical inactivity and other factors seem to be directly linked to the epidemic of non-communicable diseases in societies with these characteristics.

In addition to the interaction between UGS and biodiversity, the interaction of SES with the environmental context has also gained some relevance. Our results showed the existence of an inverse relationship between the socioeconomic deprivation index and the boys’ BMI values. Some studies conducted with children and adolescents have highlighted the fact that areas with greater material deprivation or lower levels of wealth have higher prevalence of obesity or overweight, regardless of the socioeconomic position of the household [66,67].

However, Gordon-Larsen et al. [68] disagreed when reporting that the amount of physical activity can be linked to economic factors, whereby lower socioeconomic areas in many cases are built with fewer and worse leisure areas and also involve greater distances to reach the spaces that offer PA opportunities. Furthermore, neighborhoods can be perceived as more or less safe, and consequently more or less suitable for leisure activities for young people.

According to Noonan [69], there are complex paths that link socioeconomic disadvantages with PA and overweight or childhood obesity. The disparity between the methods used in the analysis of socioeconomic deprivation could be a relevant factor when it comes to obtaining harmonious conclusions. Evidence on this topic suggests that these socioeconomic disparities in health are the result of differentiated access to built environmental and economic resources [70]; however, until now, the analysis of socioeconomic differences in terms of amounts of PA and obesity trends in adolescents has been limited [71]. Additionally, it does not seem there is a general consensus on what constitutes socioeconomic deprivation [72].

The method used for analyzing socioeconomic deprivation could be an important factor in terms of inconsistencies, since there are several existing measures, including the education of parents or guardians and household income.

Although this study does not report significant results related to PA and overweight or obesity, further studies may be important to improve knowledge in the search for an understanding of the mechanisms by which access to green spaces, greenery and the presence of species richness in urban environments influence PA and weight in adolescent populations.

## 5. Study Limitations

Studies of the determinants of urban health are complex because cities are constantly changing, resulting in different living conditions within and between cities. In this sense, the availability of green spaces can vary considerably between different urban areas. Currently, there are no standards that accurately establish the quantity or ideal characteristics of green spaces associated with benefits for human health. 

Although this study tried to fill a knowledge gap, certain limitations need to be recognized. First, since it was a cross-sectional study, it was not possible to make an inference about causal directions. Second, although self-reported anthropometrics are valuable sources of data, the self-reported bias can have important consequences for the accuracy of screening for overweight and obesity [73]. Previous studies have shown that self-reported weights are significantly lower than the measured weights [74,75]; however, the use of BMI resulting from self-reported data shows good performance, moderate sensitivity and high specificity. With that said, this appears to be an alternative when direct BMI measurement is not available [76].

Third, we had no information about possible confounding variables, such as concerns about safety, traffic or related to overall aesthetics environments’, which made impossible testing their effects. In addition, it is likely that there are other attributes of green spaces (e.g., quality, size) that we did not capture that could influence the levels of adolescent PA, and which consequently could impact BMI values.

Fourth, despite being considered an a priori estimate of the ideal sample size for this research, the smaller geographical extent of the species richness dataset forced us to conduct the study using a smaller sample. This may have influenced the results, especially with regard to the probability of detecting statistical significant associations between variables [77]. Furthermore, determining the sample size when applying multilevel models is important, as the statistical power depends on the total sample size for each level [78]. In this context, considering that statistical power and *p* values depend on both the effect size and the sample size, the sample used probably did not provide enough power to find statistical differences [77].

Despite these weaknesses and the lack of evidence on the relationships between the amount of UGS, green exposure and the amount of species richness inserted in the residential environment with PA and BMI for Portuguese adolescents, the results of this study are a relevant contribution to the literature as they provide are groundbreaking.

## 6. Conclusions

In this study, we did not find relations between the number of urban green spaces, green exposure and species richness in residence areas with PA and incidence rates of overweight and obesity among a Portuguese adolescent population. 

The relationships between the amount of UGS, exposure to green spaces and the richness of species with PA and BMI seem to be complex, and further studies are needed for a better understanding of this topic.

## Figures and Tables

**Table 1:** Sample characteristics.

		Normal Weight			Overweight and Obesity		
	Total Sample (*n* = 62)	Total Sample (*n* = 48)	Girls (*n* = 25)	Boys (*n* = 23)	Total Sample (*n* = 14)	Girls (*n* = 7)	Boys (*n* = 7)
Age (years), M ± SD	16.4 ± 1.5	16.3 ± 1.4	16.0 ± 1.5	16.6 ± 1.3	16.1 ± 1.3	16.2 ± 1.6	16.0 ± 1.1
Weight (kg), M ± SD	61.4 ± 11.1	58.3 ± 8.8	54.9 ± 8.6	61.9 ± 7.8	74.0 ± 10.2	70.3 ± 8.3	77.6 ± 11.2
Height (m), M ± SD	1.67 ± 0.10	1.67 ± 0.1	1.62 ± 0.9	1.72 ± 0.7	1.65 ± 0.1	1.6 ± 0.1	1.7 ± 0.1
BMI (kg/m^2^), M ± SD	21.9 ± 3.2	20.7 ± 2.0	20.6 ± 2.4	20.8 ± 1.6	26.9 ± 1.8	27.1 ± 2.2	26.6 ± 1.4
MVPA Average Daily (minutes), M ± SD	41.2 ± 20.8	41.5 ± 22.2	38.4 ± 184	44.1 ± 25.7	42.2 ± 16.6	42.2 ± 13.9	42.2 ± 20.1
EDI-PT, M ± SD	2.1 ± 0.8	2.19 ± 0.8	2.8 ± 0.8	2.3 ± 0.8	1.7 ± 0.8	2.1 ± 0.6	1.4 ± 0.7

BMI: body mass index; MVPA: moderate-to-vigorous physical activity; EDI-PT: Index of Deprivation for Small Portuguese Areas.

**Table 2:** Descriptive statistics for each variable by gender and by body mass index categories.

	Girls (*n* = 32)			Boys (*n* = 30)		
	Normal Weight (*n* = 25)	Overweight and Obesity (*n* = 7)	*p* (within Groups)	Normal Weight (*n* = 23)	Overweight and Obesity (*n* = 7)	*p* (within Groups)
BMI (kg/m^2^), M ± SD	20.7 ± 2.5	27.2 ± 2.2	0.71	20.8 ± 1.7	26.6 ±1.4	0.62
MVPA Average Daily (minutes), M ± SD	38.4 ± 18.5	42.2 ± 13.9	0.25	44.1 ± 25.8	42.3 ± 20.1	0.63
EDI-PT (terciles), M ± SD	2.1 ± 0.8	2.2 ± 0.7	0.37	2.3 ± 0.8	1.4 ± 0.8	0.48
UGS Counts [300 m], M ± SD	0.4 ± 0.5	0.5 ± 0.7	0.27	0.2 ± 0.4	0.7 ± 0.7	0.04 *
UGS Counts [500 m], M ± SD	1.0 ± 0.9	1.0 ± 1.1	0.53	0.8 ± 0.7	1.4 ± 1.0	0.53
UGS Counts [1000 m], M ± SD	3.4 ± 2.1	3.4 ± 3.2	0.05	3.0 ± 2.4	4.8 ± 2.6	0.80
UGS Counts [1500 m], M ± SD	6.8 ± 3.6	7.4 ± 3.9	0.64	5.8 ± 3.7	8.2 ± 3.3	0.70
NDVI [300 m], M ± SD	0.17 ± 0.04	0.15 ± 0.03	0.08	0.18 ± 0.05	0.16 ± 0.03	0.16
NDVI [500 m], M ± SD	0.18 ± 0.04	0.15 ± 0.03	0.71	0.18 ± 0.05	0.16 ± 0.02	0.36
NDVI [1000 m], M ± SD	0.18 ± 0.03	0.17 ± 0.03	0.59	0.19 ± 0.03	0.17 ± 0.01	0.18
NDVI [1500 m], M ± SD	018 ± 0.03	0.17 ± 0.03	0.41	0.19 ± 0.02	0.17 ± 0.02	0.22
SRI [300 m], M ± SD	3.4 ± 3.9	2.4 ± 2.4	0.40	3.3 ± 3.3	3.1 ± 2.8	0.33
SRI [500 m], M ± SD	3.7± 2.6	2.6 ± 2.2	0.47	3.6 ± 2.7	3.4± 2.5	0.85
SRI [1000 m], M ± SD	3.7 ± 1.5	3.1 ± 1.4	0.59	4.1 ± 1.4	3.2 ± 0.9	0.33
SRI [1500 m], M ± SD	3.7 ± 1.1	3.4 ± 1.3	0.39	4.0 ± 1.1	3.2 ± 0.5	0.06

Note: * significant difference (*p* < 0.05); BMI: body mass index; MVPA: moderate-to-vigorous physical activity; EDI-PT: Index of Deprivation for Small Portuguese Areas; UGS: urban green space; SRI: species richness index; NDVI: normalized difference vegetation index.

**Table 3:** The results of the multiple linear regression model for body mass index considering the urban green space, normalized difference vegetation index and species richness index values for the sample without adjustment.

Model	Variables				95% Confidence Interval for B			
		B	SE B	*β*	Lower Bound	Upper Bound	t	*p*
1	UGS Counts [300 m]	0.508	0.758	0.091	−1.010	2.077	0.670	0.505
	SRI [300 m]	−0.218	0.191	−0.224	−0.600	0.165	−1.138	0.260
	NDVI [300 m]	6.742	15.608	0.086	−24.502	37.986	0.432	0.667
	Constant	21.445	2.442		16.557	26.33	8.782	<0.001 **
	Model							0.582
2	UGS Counts [500 m]	−0.338	0.486	−0.094	−1.310	0.635	−0.694	0.490
	SRI [500 m]	−0.045	0.268	−0.036	−0.583	0.492	−0.169	0.866
	NDVI [500 m]	−7.598	18.662	−0.088	−44.954	28.759	−0.407	0.685
	Constant	23.976	2.833		18.305	29.647	8.463	<0.001 **
	Model							0.789
3	UGS [1000 m]	−0.038	0.226	−0.028	−0.491	0.416	−0.167	0.868
	SRI [1000 m]	−0.298	0.563	−0.124	−1.426	0.831	−0.528	0.599
	NDVI [1000 m]	10.094	30.792	0.086	−51.566	71.754	0.328	0.774
	Constant	21.443	4.817		11.798	31.088	4.452	<0.001 **
	Model							0.956
4	UGS [1500 m]	−0.023	0.203	−0.025	−0.429	0.384	−0.112	0.911
	SRI [1500 m]	−0.314	0.711	−0.105	−1.727	1.119	−0.428	0.670
	NDVI [1500 m]	11.940	37.403	0.091	−62.931	9.811	0.319	0.751
	Constant	21.216	6.725		7.755	34.577	3.155	0.033 *
	Model							0.974

Note: ** extremely significant difference (*p* < 0.001); * significant difference (*p* < 0.05); UGS: urban green space; SRI: species richness index; NDVI: normalized difference vegetation index.

**Table 4:** The results of the multiple linear regression model for body mass index considering urban green space, normalized difference vegetation index and species richness index values with adjustment.

Model	Variables				95% Confidence Interval for B			
		B	SE B	*β*	Lower Bound	Upper Bound	t	*p*
1	UGS Counts [300 m]	0.145	0.794	0.026	−1.445	1.734	0.182	0.856
	SRI [300 m]	−0.214	0.190	−0.221	−0.594	0.165	−1.132	0.263
	NDVI [300 m]	9.467	15.588	0.121	−21.749	40.682	0.607	0.546
	EDI-PT	−0.795	0.557	−0.199	−1.911	0.321	−1.427	0.159
	Constant	22.788	2.597		17.588	27.989	8.774	<0.001 **
	Model							0.410
2	UGS Counts [500 m]	−0.575	0.494	−0.160	−1.563	0.414	−1.164	0.249
	SRI [500 m]	−0.102	0.265	−0.080	−0.633	0.428	−0.385	0.701
	NDVI [500 m]	−2.541	18.502	−0.029	−39.592	34.509	−0.137	0.891
	EDI-PT	0.988	0.540	−0.248	−2.068	0.093	−1.830	0.072
	Constant	25.582	2.913		19.749	31.415	8.783	<0.001 **
	Model							0.360
3	UGS Counts [1000 m]	−0.129	0.226	−0.096	−0.581	0.324	−0.569	0.572
	SRI [1000 m]	−0.615	0.574	−0256	−1.766	0.535	−1.071	0.289
	NDVI [1000 m]	20.237	30.531	0.172	−40.924	81.398	0.663	0.510
	EDI-PT	−1.053	0.544	−0.267	−2.143	0.038	−1.934	0.058
	Constant	23.268	4.799		13.655	32.881	4.849	<0.001 **
	Model							0.405
4	UGS Counts [1500 m]	−0.064	0.201	−0.071	−0.466	0.339	−0.317	0.753
	SRI [1500 m]	−0.486	0.707	−0.167	−1.903	0.930	−0.687	0.495
	NDVI [1500 m]	14.705	36.834	0.113	−59.053	88.463	0.399	0.691
	EDI-PT	−0.894	0.593	−0.224	−1.940	0.153	−1.710	0.093
	Constant	23.527	6.753		10.005	37.049	3.484	<0.001 **
	Model							0.538

Note: ** extremely significant difference (*p* < 0.001); * significant difference (*p* < 0.05); UGS: urban green space; SRI: species richness index; NDVI: normalized difference vegetation index; MVPA: moderate-to-vigorous physical activity; EDI-PT: European Index of Deprivation for Small Portuguese Areas.

## Data Availability

The data supporting the findings of this study are available within the article and its supplementary materials.

## Supplementary Materials

The following are available online at https://www.mdpi.com/article/10.3390/ijerph18126588/s1.
