# Supplementary material for: Urban Green Spaces, Greenness Exposure and Species Richness in Residential Environments and Relations with Physical Activity and BMI in Portuguese Adolescents
Source: Int J Environ Res Public Health. 2021 Jun 18;18(12):6588. doi: 10.3390/ijerph18126588 (PMC8296418; doi:10.3390/ijerph18126588)
Supplement: Supplementary file 1 [file ijerph-18-06588-s001.zip › ijerph-1188943-supplementary.pdf]

Table S1. The results of the multiple linear regression model for physical activity with urban green space.

Model	Variables	95%confidence Interval for B						
		B	SE B	β	Lower bound	Upper bound	t	p
1	**UGS Counts [300m]**	0.484	4.585	0.014	-8.687	9.655	0.106	0.916
	**Constant**	41.198	3.345		34.508	47.889	12.317	<0.001**
	**Model**							0.916
2	**UGS Counts [500m]**	2.274	2.295	0.099	-3.616	8.165	0.772	0.443
	**Constant**	39.171	3.940		31.290	47.053	9.941	<0.001**
	**Model**							0.443
3	**UGS Counts [1000m]**	1.516	1.102	0.176	-0.690	3.722	1.375	0.174
	**Constant**	35.759	4.714		26.327	45.191	7.586	<0.001**
	**Model**							0.174
4	**UGS Counts [1500m]**	0.955	0.725	0.168	-0.495	2.406	1.318	0.193
	**Constant**	35.013	5.528		23.955	46.072	6.333	<0.001**
	**Model**							0.193

Note: **: extremely significant difference (p< 0.001); *: significant difference (p< 0.05); UGS: urban green space.

Table S2. The results of the multiple linear regression model for physical activity with species richness index.

Model	Variables	95%confidence Interval for B						
		B	SE B	β	Lower bound	Upper boun d	t	p
1	**Species Richness Index [300m]**	0.889	0.791	0.144	-0.692	2.471	1.125	0.265
	**Constant**	38.580	3.658		31.264	45.897	10.548	<0.001**
	**Model**							0.265
2	**Species Richness Index [500m]**	-0.459	1.046	-0.057	-2.551	1.633	0.439	0.663
	**Constant**	42.989	4.490		34.007	51.972	9.574	<0.001
	**Model**							0.663
3	**Species Richness Index [1000m]**	-1.479	1.965	-0.097	-5.409	2.452	-0.753	0.455
	**Constant**	46.782	7.623		31.534	62.030	6.317	<0.001**
	**Model**							0.455
4	**Species Richness Index [1500m]**	-2.924	2.362	-0.158	-7.649	1.801	-1.238	0.221
	**Constant**	52.211	2.362		33.970	70.452	5.725	<0.001**
	**Model**							0.221

Note: **: extremely significant difference (p< 0.001); *: significant difference (p< 0.05); SRI: species richness index.

Table S3. The results of the multiple linear regression model for physical activity with normalized difference vegetation index.

Model	Variables	95%confidence Interval for B						
		**B**	**SE B**	β	**Lower bound**	**Upper bound**	**t**	**p**
1	**NDVI [300m]**	44.023	64.024	0.088	-84.045	172.091	0.688	0.494
	**Constant**	33.769	11.429		10.908	56.629	2.955	0.004*
	**Model**							0.494
2	**NDVI [500m]**	-46.444	70.860	-0.084	-188.185	95.297	-0.655	0.515
	**Constant**	49.616	12.803		24.005	75.226	3.875	<0.001**
	**Model**							0.515
3	**NDVI [1000m]**	-77.509	96.781	-0.103	-271.100	116.082	-0.801	0.426
	**Constant**	55.653	17.984		19.679	91.627	3.095	0.003*
	**Model**							0.426
4	**NDVI [1500m]**	-206.847	104.237	-0.248	-415.352	1.659	-1.984	0.052
	**Constant**	79.592	19.416		40.753	118.430	4.099	<0.001**
	**Model**							0.052

Note: **: extremely significant difference (p< 0.001); *: significant difference (p< 0.05); NDVI: normalized difference vegetation index.

Table S4. The results of the multiple linear regression model for physical activity with urban green space, species richness index and normalized difference vegetation index for girls.

Model	Variables				95%confidence Interval for B			
		B	SE B	β	Lower bound	Upper bound	t	p
1	**UGS Counts [300m]**	0.813	5.207	0.029	-9.854	11.480	0.156	0.877
	**SRI [300m]**	2.541	1.343	0.528	-0.209	5.291	1.982	0.069
	**NDVI [300m]**	-232.671	141.291	-0.470	-522.092	56.750	-1.647	0.111
	**Constant**	69.937	21.793		25.297	114.577	3.209	0.003*
	**Model**							0.266
2	**UGS Counts [500m]**	0.312	3.124	0.018	-6.087	6.711	0.100	0.921
	**SRI [500m]**	3.657	1.895	0.530	-0.225	7.539	1.930	0.064
	**NDVI [500m]**	-369.957	148.634	-0.696	-674.421	-65.494	-2.489	0.019*
	**Constant**	90.709	22.307		45.015	136.403	4.066	<0.001**
	**Model**							0.112
3	**UGS Counts [1000m]**	0.038	1.567	0.005	-3.176	3.253	0.025	0.981
	**SRI [1000m]**	5.595	4.051	0.439	-2.717	13.906	1.381	0.179
	**NDVI [1000m]**	-479.847	211.992	-0.760	-914.818	-44.876	-2.264	0.032
	**Constant**	104.851	31.487		40.245	169.456	3.330	0.003
	**Model**							0.090
4	**UGS Counts [1500m]**	-0.946	1.377	-0.197	-3.768	1.875	-0.687	0.498
	**SRI [1500m]**	0.747	5.512	0.054	-10.493	12.087	0.145	0.885
	**NDVI [1500m]**	-389.221	278.557	-0.575	-959.820	181.378	-1.397	0.173
	**Constant**	113.415	45.359		20.501	206.329	2.500	0.019*
	**Model**							0.189

Note: **: extremely significant difference ($p < 0.001$); *: significant difference ($p < 0.05$); UGS: urban green space; SRI: species richness index; NDVI: normalized difference vegetation index.

Table S5. The results of the multiple linear regression model for physical activity with urban green space, species richness index and normalized difference vegetation index for boys.

Model	Variables	B	SE B	β	95%confidence Interval for B Lower bound	Upper bound	t	p
1	**UGS Counts [300m]**	-2.026	9.074	-0.046	-20.679	16.626	-0.223	0.825
	**SRI [300m]**	0.298	2.414	0.039	-4.664	5.260	0.123	0.903
	**NDVI [300m]**	61.933	158.778	0.123	-264.439	388.306	0.390	0.700
	**Constant**	32.358	24.624		-18.257	82.972	1.314	0.200
	**Model**							0.865
2	**UGS Counts [500m]**	5.157	5.766	0.180	-6.695	17.010	0.894	0.379
	**SRI [500m]**	-3.073	2.889	-0.339	-9.012	2.867	-1.063	0.297
	**NDVI [500m]**	183.439	184.609	0.326	-196.031	592.908	0.994	0.330
	**Constant**	16.823	28.325		-41.401	75.046	0.594	0.558
	**Model**							0.662
3	**UGS Counts [1000m]**	4.703	2.364	0.434	-0.786	8.932	1.723	0.097
	**SRI [1000m]**	-6.043	5.565	-0.340	-17.482	5.395	-1.086	0.287
	**NDVI [1000m]**	572.116	329.677	0.652	105.545	1249.776	1.735	0.095
	**Constant**	-55.826	54.465		-167.781	56.128	-1.025	0.315
	**Model**							0.306
4	**UGS Counts [1500m]**	0.094	2.276	0.015	-4.584	4.772	0.041	0.967
	**SRI [1500m]**	5.903	7.031	0.264	-8.549	20.354	0.840	0.409
	**NDVI [1500m]**	-390.356	398.999	-0.391	-1210.510	429.798	-0.978	0.337
	**Constant**	94.357	78.518		-67.034	255.749	1.202	0.240
	**Model**							0.629

Note: **: extremely significant difference ($p < 0.001$); *: significant difference ($p < 0.05$); UGS: urban green space; SRI: species richness index; NDVI: normalized difference vegetation index.

Table S6. The results of the multiple linear regression model for body mass index with urban green space.

Model	Variables	B	SE B	β	95%confidence Interval for B Lower bound	Upper bound	t	p
1	**UGS Counts [300m]**	-0.043	0.212	-0.026	-0.467	0.381	-0.203	0.840
	**Constant**	22.187	0.468		21.251	23.123	47.421	0.000*
	**Model**							0.840
2	**UGS Counts [500m]**	-0.014	0.050	-0.036	-0.114	0.086	-0.227	0.782
	**Constant**	22.210	0.481		21.248	23.171	46.200	0.000*
	**Model**							0.782
3	**UGS Counts [1000m]**	-0.001	0.012	-0.012	-0.025	0.022	0.090	0.929
	**Constant**	22.177	0.553		21.071	23.283	40.099	0.000*
	**Model**							0.929
4	**UGS Counts [1500m]**	0.002	0.005	0.046	-0.008	0.012	0.359	0.721
	**Constant**	21.976	0.631		20.714	23.238	34.842	0.000*
	**Model**							0.721

Note: **: extremely significant difference (p< 0.001); *: significant difference (p< 0.05); UGS: urban green space.

Table S7. The results of the multiple linear regression model for body mass index with species richness index.

Model	Variables	B	SE B	β	95%confidence Interval for B Lower bound	Upper bound	t	p
1	**Species Richness Index [300m]**	-0.152	0.124	-0.156	-0.399	0.096	-1.226	0.225
	**Constant**	22.627	0.572		21.483	23.772	39.546	0.000*
	**Model**							0.225
2	**Species Richness Index [500m]**	-0.118	0.163	-0.093	-0.721	-0.445	0.209	0.474
	**Constant**	22.551	0.702		21.147	23.955	32.130	0.000*
	**Model**							0.474
3	**Species Richness Index [1000m]**	-0.160	0.309	-0.067	-0.778	0.457	-0.520	0.605
	**Constant**	22.728	1.198		20.332	25.124	18.976	0.000*
	**Model**							0.859
4	**Species Richness Index [1500m]**	-0.027	0.375	-0.009	-0.777	0.723	-0.072	0.943
	**Constant**	22.245	1.447		19.349	25.140	15.369	0.000*
	**Model**							0.943

Note: **: extremely significant difference (p< 0.001); *: significant difference (p< 0.05); SRI: species richness index.

Table S8. The results of the multiple linear regression model for body mass index with normalized vegetation difference index.

Model	Variables	95%confidence Interval for B						
		B	SE B	β	Lower bound	Upper bound	t	p
1	**NDVI [300m]**	-7.345	10.030	-0.094	-27.407	12.717	-0.732	0.467
	**Constant**	23.420	1.790		19.838	27.001	13.081	0.000*
	**Model**							0.467
2	**NDVI [500m]**	-7.989	11.098	-0.093	- 30.188	14.211	-0.720	0.474
	**Constant**	23.557	2.005		19.545	27.568	11.748	0.000*
	**Model**							0.474
3	**NDVI [1000m]**	-2.431	15.246	-0.021	- 32.929	28.066	-0.159	0.874
	**Constant**	22.592	2.833		16.925	28.259	7.974	0.000*
	**Model**							0.874
4	**NDVI [1500m]**	3.141	16.860	0.024	-30.584	36.867	-0.186	0.853
	**Constant**	21.565	3.141		15.283	27.847	6.867	0.000*
	**Model**							0.853

Note: **: extremely significant difference (p< 0.001); *: significant difference (p< 0.05); NDVI: normalized difference vegetation index.

Table S9. The results of the multiple linear regression model for body mass index with urban green space.

species richness index and normalized vegetation difference index for girls.

Model	Variables	95%confidence Interval for B						
		B	SE B	β	Lower bound	Upper bound	t	p
1	**UGS Counts [300m]**	0.167	0.471	0.099	-0.798	1.132	0.354	0.726
	**SRI [300m]**	-0.460	0.426	-0.461	-1.333	0.413	-1.079	0.290
	**NDVI [300m]**	20.570	35.943	0.200	-53.056	94.196	0.572	0.572
	**Constant**	19.931	5.265		9.147	30.714	3.786	0.001*
	**Model**							0.533
2	**UGS Counts [500m]**	-0.056	0.105	-0.142	-0.271	0.159	-0.532	0.599
	**SRI [500m]**	-0.024	0.610	-0.017	-1.873	1.225	-0.040	0.969
	**NDVI [500m]**	-6735	39.966	-0.061	-88.602	75.132	-0.169	0.867
	**Constant**	23.660	5.604		12.182	35.139	4.222	0.000*
	**Model**							0.829
3	**UGS Counts [1000m]**	-0.005	0.020	-0.050	-0.046	0.036	-0.243	0.810
	**SRI [1000m]**	-0.193	0.941	-0.073	-2.121	1.736	-0.205	0.839
	**NDVI [1000m]**	18.892	49.535	0.143	-82.577	120.360	0.381	0.706
	**Constant**	19.563	6.664		5.912	33.214	2.935	0.007*
	**Model**							0.939
4	**UGS Counts [1500m]**	0.005	0.010	0.114	-0.015	0.025	0.503	0.619
	**SRI [1500m]**	-0.507	1.217	-0.166	-3.001	1.987	-0.416	0.680
	**NDVI [1500m]**	53.749	60.720	0.383	-70.631	178.128	0.885	0.384
	**Constant**	13.717	8.154		-2.986	30.421	1.682	0.104
	**Model**							0.744

Note: **: extremely significant difference (p< 0.001); *: significant difference (p< 0.05); UGS: urban green space; SRI: species richness index NDVI: normalized difference vegetation index.

Table S10. The results of the multiple linear regression model for body mass index with urban green space, species richness index and normalized difference vegetation index for boys.

Model	Variables	95%confidence Interval for B						
		B	SE B	β	Lower bound	Upper bound	t	p
1	UGS Counts [300m]	0.347	0.416	0.217	-0.508	1.201	0.834	0.412
	SRI [300m]	-0.133	0.372	-0.143	-0.897	0.631	-0.359	0.723
	NDVI [300m]	3.077	22.151	0.050	-42.455	48.609	0.139	0.891
	Constant	21.758	3.227		15.022	28.494	6.639	0.000*
	Model							0.830
2	UGS Counts [500m]	-0.068	0.093	0.177	-0.122	0.259	0.738	0.467
	SRI [500m]	-0.059	0.432	-0.053	-0.947	0.830	-0.136	0.893
	NDVI [500m]	-3.686	25.266	-0.054	-55.621	48.249	-0.146	0.885
	Constant	22.804	3.559		15.488	30.121	6.407	0.000*
	Model							0.810
3	UGS Counts [1000m]	0.002	0.020	0.022	-0.039	0.042	0.092	0.927
	SRI [1000m]	-0.579	0.710	-0.268	-2.038	0.879	-0.816	0.422
	NDVI [1000m]	4.864	40.031	0.046	-77.420	87.149	0.122	0.904
	Constant	23.429	6.070		10.952	35.907	3.860	0.001*
	Model							0.666
4	UGS Counts [1500m]	0.002	0.010	0.053	-0.018	0.022	0.199	0.844
	SRI [1500m]	-0.388	0.853	-0.143	-2.140	1.365	-0.455	0.653
	NDVI [1500m]	-4.375	45.393	-0.036	-97.682	88.931	-0.096	0.924
	Constant	24.318	7.271		9.372	39.264	3.344	0.003*
	Model							0.761

Note: **: extremely significant difference (p< 0.001); *: significant difference (p< 0.05); UGS: urban green space; SRI: species richness index; NDVI: normalized difference vegetation index.
